# Clinical and epidemiological profile of patients with definite ocular sarcoidosis at a Brazilian referral center

**DOI:** 10.5935/0004-2749.2022-00131

**Published:** 2025-02-11

**Authors:** Eduardo Ferracioli-Oda, Alexandre de Melo Kawassaki, Fábio Eiji Arimura, Ronaldo Adib Kairalla, Carlos Eduardo Hirata, Joyce Hisae Yamamoto

**Affiliations:** 1 Department of Ophthalmology, LIM-33, Hospital das Clínicas, Faculdade de Medicina, Universidade de São Paulo, São Paulo, SP, Brazil; 2 Pulmonary Division, Instituto do Coração, Faculdade de Medicina, Universidade de São Paulo, São Paulo. SP, Brazil

Dear Editor,

Sarcoidosis is a multisystem inflammatory disease of unknown etiology, with an annual incidence ranging from 1 to 121 per 100,000 individuals^(1)^. Intraocular involvement occurs in 30%-60% of patients with systemic sarcoidosis^(2,3)^. In Brazil, the incidence is less than 10/100,000^4^. In Hospital das Clinicas HCFMUSP, Faculdade de Medicina, Universidade de São Paulo, the largest teaching hospital and referral center in Brazil, sarcoidosis was identified as the etiology in 2.3% of cases of uveitis^(4)^.

The revised criteria of the International Workshop on Ocular Sarcoidosis (IWOS) were published in 2019^(5)^. To our knowledge, no study has applied these criteria in Brazilian patients with ocular sarcoidosis. We aimed to characterize the clinical and epidemiological profile of 15 patients with biopsy-confirmed ocular sarcoidosis treated at a Brazilian referral center and to evaluate the applicability of the revised IWOS diagnostic criteria^(3)^. Data were collected from January 2018 to April 2019 and comprised a cross-checked database of the Department of Ophthalmology (HCFMUSP) and the Pulmonary Division of the Heart Institute (InCor, FMUSP). Other causes of uveitis and orbital inflammation were excluded during follow-up. The Institutional Ethics Committee approved the study protocol (CapPesq 3.972.915).

There were 11 female patients (73.3%). Eight patients (53.3%) were older than 60. The mean age at diagnosis was 53.0 ± 14.6 years. Nine patients were White (60%), five were Black or mixed-race (33.3%), and one was Asian (6.7%). Ocular involvement was 2 years before systemic diagnosis in one patient (6.7%), concomitant in eight patients (53.3%), and was later in six patients (40%), with a mean interval of 2.5 ± 1.2 years. Thirteen patients had uveitis (86.7%), and one had orbital or lacrimal gland involvement. Biopsy samples were collected from the lung (n=11, 73.4%), skin (n=5, 33.4%), lacrimal gland (n=1, 6.6%) and orbital tissue (n=1, 6.6%). Thirteen patients (86.6%) received oral prednisone alone or combined with other immunosuppressants. In 17 eyes (60.7%), visual acuity stabilized or improved after treatment. Eleven of 30 eyes (36.6%) experienced deteriorating visual acuity (>0.2 logMAR) during the follow-up due to cataracts in four eyes (13.3%), glaucoma in four eyes (13.3%), epiretinal membrane and macular edema in one eye (3.3%), a macular chorioretinal scar in one eye (3.3%), and rhegmatogenous retinal detachment in one eye (3.3%). All epidemiological and clinical data are detailed in table 1.

**Table 1:** Epidemiological and clinical data of patients with definite ocular sarcoidosis (n=15)

Age at diagnosis, years, mean (median, range)		53	(60; 29-78)
Ethnicity, n (%)			
White		9	(60)
Black		3	(20)
Mixed ethnicity		2	(13.3)
Asian		1	(6.7)
Sex, n (%)			
Female		11	(73.3)
Male		4	(26.7)
Follow-up time, years, mean (median, range)		12.2	(12; 1-33)
Type of ocular involvement, n (%)			
Orbital inflammatory disease		1	(6.6)
Lacrimal gland infiltration		1	(6.6)
Uveitis			
Anterior		5	(33.4)
Intermediate		2	(13.4)
Posterior		1	(6.6)
Diffuse		5	(33.4)
Biopsy tissue, n (%)^a^			
Lung		11	(73.4)
Skin		5	(33.4)
Orbital tissue		1	(6.6)
Lacrimal gland		1	(6.6)
Type of treatment, n (%)			
Topical only		1	(6.6)
Oral prednisone		3	(20)
Oral prednisone + subtenon triamcinolone injection		1	(6.6)
Oral prednisone + methotrexate		7	(46.8)
Oral prednisone + methotrexate + leflunomide or infliximab		3	(20)
Visual acuity, logMAR (n=28), n of eyes (%)^b^			
Baseline	<0.3	17	(60.7)
	>0.3	11	(39.1)
Last evaluation	<0.3	14	(50)
	>0.3	14	(50)
Change in visual acuity from first to last evaluation (n=28), n of eyes (%)^c^	Worsening	11	(39.3)
	Stable	12	(42.8)
	Improvement	5	(17.9)

The revised IWOS criteria were applied in the 13 patients with uveitis that were classified as anterior (n=5, 33.4%), intermediate (n=2, 13.4%), posterior (n=1, 6.6%), and diffuse (n=5, 33.4%). Both eyes were affected in all patients; mutton-fat keratic precipitates or iris nodules were observed in seven patients (53.9%), while posterior segment signs were present in six patients (46.1%). Six patients (46.1%) had three or more clinical signs. Concerning radiological signs, bilateral hilar lymphadenopathy (BHL) was present in 12 patients (92.3%); the patient without BHL had parenchymal lung abnormalities consistent with sarcoidosis. The tuberculin test was negative in all tested patients. The IWOS criteria are shown in table 2.

**Table 2:** Distribution of the revised IWOS criteria in patients with uveitis diagnosed as definite ocular sarcoidosis (n=13)

Description			
Clinical signs	n (%)		eyes (%)
Bilaterality		13 (100)	
Mutton-fat keratic precipitates/iris nodules		7 (53.9)	14(53.9)
Tent-shaped peripheral anterior synechiae/trabecular meshwork nodules		2(15.4)	3 (11.5)
Snowballs/string-of-pearls vitreous opacity		3 (23.1)	6(23.1)
Multiple chorioretinal peripheral lesions		3 (23.1)	6(23.1)
Nodular/segmental periphlebitis or microaneurysm in inflamed eye		4 (30.8)	8 (30.8)
Optic disc nodule(s)/granuloma(s) and/or solitary choroidal nodule		0	
Systemic findings, n (%)			
Bilateral hilar lymphadenopathya		12 (92.3)	
Parenchymal lung abnormalities consistent with sarcoidosisa		10(76.9)	
Tuberculin test or IGRA	Negative Positive Not performed	10(76.9) 0 3 (23.1)	
Elevated serum ACE	Not performed	-	
Elevated lysozyme Not performed		-	
Elevated CD4/CD8 ratio (> 3.5) in bronchoalveolar lavage fluid	Elevated Normal Not performed	2(15.4) 1 (7.7) 10(76.9)	
Abnormal accumulation on gallium-67 scintilography or PET-CT scan	Abnormal Not performed	1 (7.7) 12 (92.3)	
Lymphopenia		3 (23.1%)	

KP= keratic precipitates; IGRA= interferon-gamma release assay; ACE= angiotensin converting enzyme; PET-CT= positron emission tomography-computed tomography.

a = Chest CT scan performed in 12 of 13 patients: chest X-ray imaging was the only radiological investigation in 1 patient.

Our sample was predominantly White and female, with an average of 53 years, similar to other studies^(2,3)^. Concerning the IWOS diagnosis criteria, bilaterality was the single ocular clinical criterion observed in all patients with uveitis, followed by the presence of mutton-fat keratic precipitates or iris nodules. Acharya et al. completed a multicenter study of 884 patients with uveitis from 12 countries and produced similar results among 98 patients with biopsy-proven sarcoidosis^(1)^. Bilaterality was the main clinical sign (86%, compared to 100% in our sample), and BHL was observed in 81% of those patients (92.3% compared to our sample). Sixty-two patients (63%) had three or more clinical criteria, higher than the 46.1% observed in our study.

Our results are limited by the small sample size and the retrospective study design. The applicability of the revised IWOS diagnostic criteria was limited because the systemic investigation, including angiotensin-converting enzyme or lysozyme testing, CD4/CD8 ratio in bronchoalveolar lavage, and scintigraphy or positron emission tomography-computed tomography (PET-CT) imaging, were not available for most patients. In addition, as tissue biopsies are considered a more straightforward diagnostic approach, the clinician considered exams, such as bronchoalveolar lavage, unnecessary.

In conclusion, we described the clinical and epidemiological profile of 15 Brazilian patients with biopsyconfirmed ocular sarcoidosis. Bilateral involvement was universal in patients with uveitis ^(2,3)^. Further, even though the revised IWOS diagnostic criteria could not be completely analyzed, our findings support the role of radiological and tuberculin testing/interferon-gamma release assay (IGRA) as important complementary diagnostic investigations. Systemic investigations are recommended in cases with suggestive ocular findings or, when available, a diagnostic biopsy could be used to achieve a straightforward diagnosis.
